# Effects of Virgin Olive Oils Differing in Their Bioactive Compound Contents on Metabolic Syndrome and Endothelial Functional Risk Biomarkers in Healthy Adults: A Randomized Double-Blind Controlled Trial

**DOI:** 10.3390/nu10050626

**Published:** 2018-05-16

**Authors:** Estefania Sanchez-Rodriguez, Elena Lima-Cabello, Sara Biel-Glesson, Jose R. Fernandez-Navarro, Miguel A. Calleja, Maria Roca, Juan A. Espejo-Calvo, Blas Gil-Extremera, Maria Soria-Florido, Rafael de la Torre, Montserrat Fito, Maria-Isabel Covas, Juan de Dios Alche, Emilio Martinez de Victoria, Angel Gil, Maria D. Mesa

**Affiliations:** 1 Department of Biochemistry and Molecular Biology II, Institute of Nutrition and Food Technology “José Mataix”, Biomedical Research Center, University of Granada, Parque Tecnológico de la Salud, Avenida del Conocimiento s/n, 18100 Armilla, Granada, Spain; estefaniasr@outlook.com (E.S.-R.); mdmesa@ugr.es (M.D.M.); 2 Department of Biochemistry, Cell and Molecular Biology of Plants, Estación Experimental del Zaidín (CSIC), Profesor Albareda 1, 18008 Granada, Spain; elimacabello@gmail.com (E.L.-C.); juandedios.alche@eez.csic.es (J.d.D.A.); 3 Fundación Pública Andaluza para la Investigación Biosanitaria de Andalucía Oriental “Alejandro Otero” (FIBAO), Avenida de Madrid 15, 18012 Granada, Spain; sbiel@fibao.es (S.B.-G.); estrategia@innofood.es (J.R.F.-N.); mangel.calleja.sspa@juntadeandalucia.es (M.A.C.); 4 Food Phytochemistry Department, Instituto de la Grasa, Consejo Superior de Investigaciones Científicas (CSIC), University Campus Pablo de Olavide, 41013 Sevilla, Spain; mroca@ig.csic.es; 5 Instituto para la Calidad y Seguridad Alimentaria (ICSA), Avenida de la Hispanidad 17, 18320 Santa Fe, Granada, Spain; jaespejo@hotmail.com; 6 Department of Medicine, University of Granada, Avenida de la Investigación 11, 18071 Granada, Spain; blasgil@ugr.es; 7 Cardiovascular Risk and Nutrition Research Group, Hospital del Mar Medical Research Institute (IMIM), Dr. Aiguader 88, 08003 Barcelona, Spain; mariat.soria@gmail.com (M.S.-F.); mfito@imim.es (M.F.); 8 Integrative Pharmacology and Systems Neuroscience Research Group, IMIM (Hospital del Mar Research Institute), Universitat Pompeu Fabra (CEXS-UPF), Dr. Aiguader 88, 08003 Barcelona, Spain; rtorre@imim.es; 9 Spanish Biomedical Research Networking Centre, Physiopathology of Obesity and Nutrition (CIBEROBN), Instituto de Salud Carlos III, Monforte de Lemos 3-5, 28029 Madrid, Spain; maria.nuproas@gmail.com; 10 NUPROAS Handelsbolag, Nackã, Sweden, NUPROAS HB, Apartado de Correos 93, 17242 Girona, Spain; 11 Department of Physiology, Institute of Nutrition and Food Technology “José Mataix”, Biomedical Research Center, University of Granada, Parque Tecnológico de la Salud, Avenida del Conocimiento s/n, 18100 Armilla, Granada, Spain; emiliom@ugr.es

**Keywords:** olive oil, virgin olive oil, olive oil polyphenols, maslinic acid, oleanolic acid, cardiovascular diseases, endothelial function, phenolic compounds, triterpenes, metabolic syndrome

## Abstract

The aim of this study was to evaluate the effect of virgin olive oils (VOOs) enriched with phenolic compounds and triterpenes on metabolic syndrome and endothelial function biomarkers in healthy adults. The trial was a three-week randomized, crossover, controlled, double-blind, intervention study involving 58 subjects supplemented with a daily dose (30 mL) of three oils: (1) a VOO (124 ppm of phenolic compounds and 86 ppm of triterpenes); (2) an optimized VOO (OVOO) (490 ppm of phenolic compounds and 86 ppm of triterpenes); and (3) a functional olive oil (FOO) high in phenolic compounds (487 ppm) and enriched with triterpenes (389 ppm). Metabolic syndrome and endothelial function biomarkers were determined in vivo and ex vivo. Plasma high density lipoprotein cholesterol (HDLc) increased after the OVOO intake. Plasma endothelin-1 levels decreased after the intake of the three olive oils, and in blood cell cultures challenged. Daily intake of VOO enriched in phenolic compounds improved plasma HDLc, although no differences were found at the end of the three interventions, while VOO with at least 124 ppm of phenolic compounds, regardless of the triterpenes content improved the systemic endothelin-1 levels in vivo and ex vivo. No effect of triterpenes was observed after three weeks of interventions. Results need to be confirmed in subjects with metabolic syndrome and impaired endothelial function (Clinical Trials number NCT02520739).

## 1. Introduction

Metabolic syndrome (MS) is a cluster of associated metabolic and clinical disturbances that tend to occur together [1]. This syndrome is commonly represented by the combination of obesity (particularly abdominal), hyperglycemia, dyslipidemia, and hypertension [2]. MS is associated with an increased risk of cardiovascular disease (CVD), which is the main cause of disability and mortality in industrialized countries, and is associated with a chronic inflammatory response characterized by abnormal cytokine production leading to endothelial dysfunction [3].

There is consolidated clinical evidence that the Mediterranean diet (MD) is associated with a lower risk of CVDs, including myocardial infarction, stroke and cardiovascular death [4]. Mayneris-Perxachs et al. [5] proposed the MD as a successful tool for the prevention and treatment of MS and related comorbidities. A large study in a high cardiovascular risk population has found that the MD supplemented with virgin olive oil (VOO) protects people from vascular disease, suggesting a key role of olive oil [6]. A recent systematic review and meta-analysis provided evidence that olive oil might exert beneficial effects on endothelial function and biomarkers of inflammation, thus representing a key ingredient contributing to the MD cardiovascular-protective effects [7]. Olive oil is not only a source of monounsaturated fatty acids (MUFAs) but also an important source of bioactive compounds, such as phenols and triterpenes [8,9]. Previous studies suggested a protective effect of olive oil phenolic compounds on endothelial dysfunction [10], whereas olive oil triterpenes could be useful for the prevention of multiple diseases related to cell oxidative damage [11]. Maslinic and oleanolic acids are the principal triterpenes found in VOO. The potential of these olive oil triterpenic acids for use as a therapeutic strategy to improve vascular function and treating CVD has been recently reviewed [12]. However, to our best knowledge, no clinical trial has been performed to provide evidence of their benefits in healthy adults, according to EFSA requirement [13]. The present study which acronyms is NUTRAOLEUM, aimed to evaluate the effect of VOO enriched with bioactive compounds, such as phenolic compounds and triterpenes, on MS and endothelial function biomarkers in healthy adults. We reported that daily intake of VOO enriched in phenolic compounds during three weeks improved plasma high density lipoprotein cholesterol levels (HDLc), one of the features of metabolic syndrome, although no differences were found at the end of the three interventions. In addition, VOO with at least 124 ppm of phenolic compounds, regardless of the triterpenes content, improved the systemic endothelin-1 levels in vivo and ex vivo, while no additional effect of triterpenes was observed.

## 2. Materials and Methods

### 2.1. Subjects

**Figure 1:**
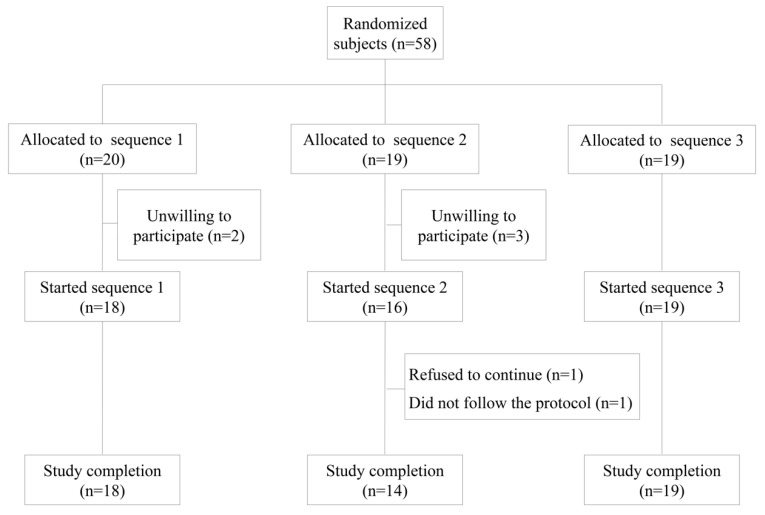
CONSORT Based Flow Diagram of the recruitment, enrollment and randomization process.

Information about subjects, sample size and their eligibility and dietary control have been referenced in detail elsewhere [14]. In brief, fifty-eight intention-to-treat subjects were eligible. The inclusion criteria were as follows: people in good health on the basis of a physical examination and basic biochemical and hematological analyses, and willingness to provide written informed consent. The exclusion criteria were as follows: smoking, intake of antioxidant supplements, aspirin or any other drug with established antioxidant properties, hyperlipidemia, obesity (body mass index (BMI) >30 kg/m^2^), diabetes, hypertension, celiac or other intestinal disease, any condition limiting mobility, life-threatening diseases, or any other disease or condition that would impair compliance. Five subjects declined to participate for personal reasons before olive oil type allocation. Fifty-three subjects (27 men and 26 women) aged from 20 to 50 years from the general population of Granada were enrolled in the study from February 2014 to July 2014 and were assigned into groups. Two subjects did not complete the study. After the first intervention, one subject refused to continue for personal reasons, and the other did not follow the protocol correctly. At the end of the experimental period, 51 subjects remained in the study (Figure 1) [14]. All subjects provided written informed consent according to the principles of the Declaration of Helsinki, and the local institutional review board, the Ethics Committee Research Centre of Granada, approved the protocol (13/11 C38).

### 2.2. Study Design

The NUTRAOLEUM study has been designed to evaluate the effects of VOO high in phenolic compounds and enriched with triterpenes, maslinic and oleanolic acids, from olive exocarp, on MS features and endothelial function risk biomarkers in comparison with a standard VOO. The characteristics of the olive oils used in the study, the design of the study, and the detailed study procedures of the sustained consumption study have been previously published [14]. In brief, the trial was a randomized, crossover, controlled and double-blind clinical trial involving three oils: (1) an optimized VOO high in phenolic compounds (OVOO) (490 ppm of phenolic compounds and 86 ppm of triterpenes) produced from Picual olives (Andalucía, Spain); (2) a functional olive oil (FOO), that was the same OVOO high in phenolic compounds (487 ppm) and enriched with triterpenes (389 ppm) from olive exocarp; and (3) a VOO obtained from the OVOO after washing to eliminate the majority of phenolic compounds (124 ppm of phenolic compounds and 86 ppm of triterpenes). San Francisco de Asís Cooperative (Montefrío (Granada), Spain) provided the olive oils used in the present study. Daily doses of 30 mL of the three types of raw olive oils, as recommended by the US Food and Drug Administration [15], were distributed over three meals. Olive oils were blindly prepared in special containers; the three types of olive oil were labeled “A”,”B”, and “C”. Containers with the corresponding 30 mL olive oil daily dose and enough amount of the same oil for cooking during each intervention period were delivered to the subjects at the beginning of each intervention period. The subjects were randomly assigned to three orders of administration of olive oil, paired by gender and age, using the block-randomization method of a software program for sequence generation [14]. The randomization lists were concealed in a lightproof sealed envelope. The sealed envelopes were kept by the independent statistician during the study, avoiding the breaking of the seal. Thus, the subjects, investigators, and outcome assessors were blinded and could not foresee the treatment allocation throughout the study. Hence, blinding of outcome assessment was also ensured. According to previous published studies, olive oils were sequentially administered over three periods of 3 weeks [16] preceded by two weeks of washout periods [17] during which the subjects were requested to avoid olives and olive oil consumption. A nutritionist personally advised subjects on replacing all types of habitually consumed raw fats using only the assigned oil. The advantage of using a crossover design is that each subject served as his or her own control. Olive oils were specially prepared for the trial and differed only in phenolic compound and triterpenes contents (Table 1). The oils were prepared in dark, sealed containers and were similar in appearance and color, thus ensuring blinding of the subjects and study personnel. The trial has been registered at ClinicalTrials.gov ID: NCT02520739.

### 2.3. Evaluation of Dietary Intake

Subjects completed a 3-day dietary record at baseline and during each intervention period [18]. Energy consumption and dietary intakes of macro- and micronutrients data were processed using CSG software (General ASDE) and the Spanish Food Composition Database (BEDCA) for the subjects who completed the intervention [19].

### 2.4. Blood Sample Collection

Fasting venous blood samples were collected at the beginning of the study (baseline) and before (pre-intervention, after the washout period) and at the end (post-intervention) of each olive oil intervention period using EDTA-coated tubes. Three-milliliter aliquots were stored at 4 °C for the ex vivo experiments. The rest of the blood samples were centrifuged (4 °C, 10 min at 1750× *g*), and plasma aliquots were immediately frozen and stored at −80 °C until analysis.

### 2.5. Ex-Vivo Whole Blood Cultures

An aliquot of blood samples (as indicated above) were collected using lithium–heparin tubes (BD Vacutainer System, Heidelberg, Germany) from a subsample of 36 subjects. Blood was diluted 1:3 with Dulbecco’s modified Eagle’s medium and agitated gently in 3-mL tubes (Greiner Bio-one, Solingen, Germany) within 3 h after collection. One-milliliter aliquots were seeded in each well of 24-well plates (Nunc, VWR International GmbH, Langenfeld, Germany) and cultured for 24 h at 37 °C under an atmosphere of 5% CO_2_. From each blood drawing, we performed triplicate incubations in parallel with positive and negative controls, separate cultures that included phytohaemagglutinin (PHA, 10 μg/mL), *E. coli* lipopolysaccharide (LPS, 1 μg/mL) and phorbol 12-myristate 13-acetate plus ionomycin (PMA, 25 ng/mL + IO, 1 μg). The same lots of PHA, LPS, PMA + IO and phosphate buffered saline were used in all experiments. Blood cultures were removed from each well and centrifuged at 700× *g* for 5 min at 20 °C. The resulting supernatants (plasma) were aliquoted and pooled from eight subjects from each of the three assigned orders of administration of olive oil and stored at −20 °C until further analysis of endothelin-1 [20,21].

### 2.6. Measurement of Metabolic Syndrome Biomarkers

In the fasting state, anthropometric measurements (weight, height and waist circumference) were determined at baseline and before and after each intervention period by the same member of the professional staff. For all measurements, the subjects did not wear shoes. MS biomarkers were determined as primary outcomes. BMI was calculated as weight (kg) divided by height squared (m^2^). Total cholesterol, triacylglycerols and serum glucose were determined by standard enzymatic methods using a PENTRA-400 autoanalyzer (ABX-Horiba Diagnostics, Montpellier, France). Plasma HDLc was measured as soluble HDLc as determined using an accelerator selective detergent method (ABX-Horiba Diagnostics). Plasma low density lipoprotein cholesterol (LDLc) concentrations were calculated using the Friedewald formula. Systolic (SBP) and diastolic (DBP) blood pressures were measured with a mercury sphygmomanometer after a minimum of 10 min resting in the seated position; the average of two measurements was recorded. The pulse pressure was calculated as the difference between SBP and DBP. Total cholesterol, triacylglycerols, serum glucose, HDLc and LDLc could only be measured for 46 subjects.

### 2.7. Measurement of Selected Plasma Hormones and Endothelial Function Biomarkers

A Milliplex Map Kit, human monoclonal antibody kits (EMD Millipore Corporation, Billerica, MA, USA) were used according to the manufacturer’s instructions in conjunction with a Luminex^®^ 200 system with the XMap technology (Luminex Corporation, Austin, TX, USA) to determine the concentrations of the following biomarkers as secondary outcomes: adiponectin (coefficient of variation (CV): 10.3%), resistin (CV: 7.7%), soluble intercellular adhesion molecule (sICAM-1) (CV: 6.1%) and soluble vascular adhesion molecule (sVCAM-1) (CV: 5.4%), (Cat. #HADK1MAG-61K).

Endothelin-1 was determined as a secondary outcome by ELISA (CV): 7.2%) (R&D Systems, Minneapolis, MN, USA; Cat. DET100) in both plasma and whole blood culture supernatants.

### 2.8. Measurements of Triterpenes and Phenolic Compounds in Urine

To ensure the subjects’ compliance with the assigned intervention, triterpenes (maslinic and oleanolic acid) derivatized with 2-picolylamine (see Appendix A) and olive oil phenolic compounds (hydroxytyrosol and metabolites) were analyzed in 24-h urine from 12 random subjects by liquid chromatography coupled to a mass spectrometer [22].

### 2.9. Statistical Analysis

Baseline data are presented as the mean values ± standard error of the mean (SEMs) unless otherwise indicated. The normality of variables was assessed using Q-Q graphs. The χ^2^ test was used for categorical variables to determine differences in the baseline. One-factor ANOVA or Kruskal-Wallis tests were used (depending on whether the normality assumption was met) for continuous variables to determine differences among the three olive oil interventions, in terms of baseline characteristics, nutrient intake and for the ex vivo endothelin-1 experiment.

Biochemical parameters are presented as adjusted mean values ± SEMs and were analyzed using a linear mixed-effects model (LMM). The normality of the residues was evaluated using Q-Q graphs. Missing data were imputed using appropriate methods. The outliers for each intervention were removed if kurtosis > 1 and asymmetry > 1 in the distribution of the responses. In all cases, more than 80% of the data were analyzed.

Variables with a skewed distribution were logarithm-transformed for analysis (nutritional variables and resistin). A LMM was used to compare variables before and after each intervention (pre- vs. post-interventions, intra-treatment effect) and to compare the results between the groups after the 3-week intervention (inter-treatment effect), adjusting for age, gender, pre-intervention and period as fixed effects and for subjects and hospital as random effects. The same model was also used to compare changes of the variables (post-intervention minus pre-intervention) without adjusting for pre-intervention. Carryover effects were assessed as the interaction between period and intervention [23]. The multiple comparison post hoc is given by the estimated means in the model (adjusted by Sidak). This statistical model (LMM) takes into account all the possible confounders (covariates) which are included on it. In addition, the baselines are used as outcomes but without effect, so they can act as a control and take into account a possible carryover effect [24]. Within the LMMs, the factor treatment, time and the random effect considered by participant are taken into account in the structure of the data.

In addition, an interaction term was checked for differences on the effect part intervention by gender. Model goodness-of-fit was tested using residual plots. The Bayesian Information Criterion was used to assess model reduction and the selection of variables and interactions. We performed all analysis on an intention-to-treat basis. A *p* < 0.05 value was considered significant. Statistical Package for the Social Sciences version 20 software was used to perform the statistical analysis (SPSS Inc., Chicago, IL, USA).

## 3. Results

### 3.1. Baseline Characteristics

Table 2 and Table 3 show the clinical and biochemical characteristics, and the average daily nutritional intakes, respectively, of the subjects grouped according to the sequence of olive oil administration at the beginning of the study. No differences were observed among the three groups of subjects at baseline.

### 3.2. Nutritional Analysis

Table 4 shows the average daily energy and selected nutrient intakes after the three olive oil interventions. No differences were observed among the three interventions (Table 4).

### 3.3. Plasma Metabolic Syndrome and Endothelial Function Biomarkers

Table 5 shows MS and endothelial function biomarker data before and after the three interventions. All clinical and biochemical MS biomarkers were within normal values at the beginning and at the end of the study. BMI, waist circumference, pulse pressure, and fasting plasma glucose, adiponectin and resistin concentrations were unchanged during the study.

When comparing pre- vs. post-intervention data (intra-treatment effect), HDLc levels significantly increased only after the OVOO intervention (*p =* 0.041), and only in females (*p =* 0.005). However, no differences were observed between the three interventions. Total cholesterol increased after the FOO intervention (*p =* 0.021). LDLc was unaffected. Fasting plasma triacylglycerol concentrations increased after the VOO and OVOO interventions (*p =* 0.037 and *p =* 0.002, respectively) but not after the FOO intervention. However, plasma triacylglycerols were low at the beginning of the study (78 ± 5 mg/dL). On the other hand, SBP decreased after the VOO intervention (*p =* 0.019) and increased after the FOO intervention (*p =* 0.004), while DBP and pulse pressure were unchanged after the three interventions. Plasma endothelin-1 concentrations decreased after the VOO, OVOO, and FOO interventions (*p =* 0.006, *p =* 0.006 and *p =* 0.014, respectively), and the plasma concentrations of sICAM-1 and sVCAM-1 were unchanged after the three interventions.

When analyzing the inter-treatment effects, LDLc levels were higher after the FOO intervention compared with the OVOO intervention (*p =* 0.033), and SBP was higher after the FOO intervention compared with the VOO intervention (*p =* 0.001).

The changes in metabolic clinical variables and endothelial function biomarkers (Supplementary Table S1) were similar in all the subjects after the three interventions except SBP, which increased up to 118 mmHg after the FOO but decreased after the VOO and OVOO interventions (up to 115 and 116 mmHg, respectively) (*p <* 0.001). The results show differences by gender for all interventions. However, no interactions were observed between gender and intervention.

### 3.4. Plasma Endothelin-1 Ex Vivo Experiments

Before the three interventions, pooled blood cultures challenging with PHA, LPS, or PMA + IO induced a potent increase in the supernatant concentrations of endothelin-1 in whole blood cultures.

**Figure 2:**
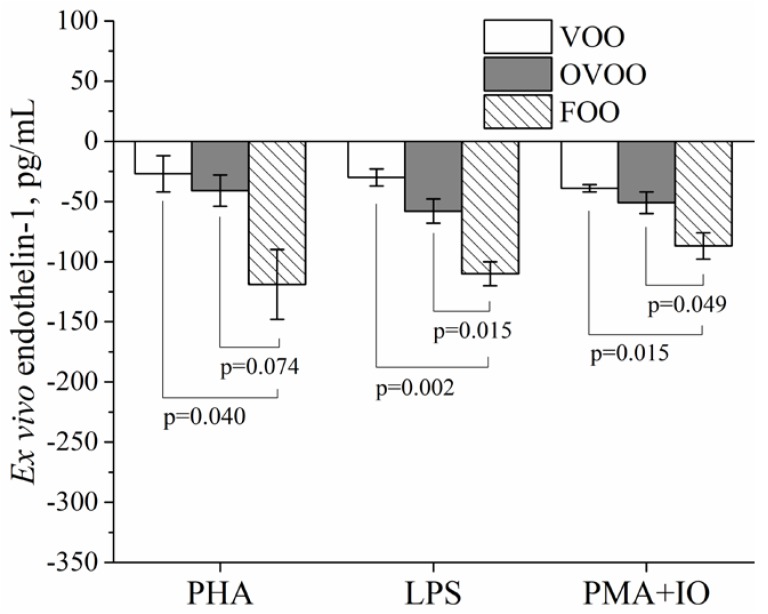
Plasma endothelin-1 ex vivo changes (post-intervention minus pre-intervention data) when stimulated with PHA, LPS or PMA + IO in whole blood cultures from healthy adults. Values are expressed as the means ± SEMs. ANOVA was used to compare differences between interventions and induction treatments. The Tukey post-hoc test was used for multiple comparisons among groups. *p <* 0.05 was considered significant. FOO, functional olive oil; IO, ionomycin; OVOO, optimized virgin olive oil; PHA, phytohemagglutinin; PMA, phorbol 12-myristate 13-acetate; VOO, virgin olive oil.

Figure 2 shows that the supernatant endothelin-1 concentration changes (post-intervention minus pre-intervention data) in whole blood cultures from the subjects were similar after the VOO and OVOO interventions and were significantly lower after the FOO intervention: −26.7 ± 15.3 pg/mL, −41.0 ± 12.9 pg/mL, and −119.5 ± 28.5 pg/mL for the VOO, OVOO and FOO interventions, respectively (*p =* 0.035), when stimulating with PHA; −30.3 ± 6.5 pg/mL, −58.2 ± 10.1 pg/mL and −109.6 ± 9.7 pg/mL for the VOO, OVOO, and FOO interventions, respectively, when stimulating with LPS (*p =* 0.002); and −38.9 ± 3.4 pg/mL, −50.8 ± 8.5 pg/mL and −87.0 ± 11.1 pg/mL for the VOO, OVOO, and FOO interventions, respectively, when stimulating with PMA+IO (*p =* 0.015).

Supplemental Figure S1 shows the supernatant endothelin-1 concentrations that were induced ex vivo with PHA, LPS, and PMA + IO in whole blood cultures from the subjects before and after the three interventions. After the VOO, OVOO, and FOO interventions, challenging with LPS or PMA + IO induced a significantly lower increase of endothelin-1 secretion in whole blood cultures, while challenging with PHA induced a significantly lower increase of endothelin-1 secretion only after the OVOO and FOO interventions.

### 3.5. Biomarkers of Intervention Compliance

**Figure 3:**
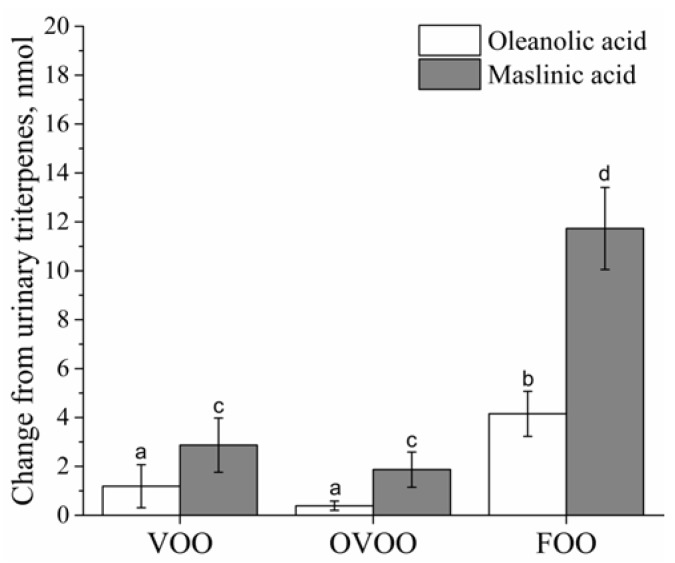
In vivo urinary triterpenes changes (post-intervention minus pre-intervention data) for each olive oil intervention in healthy adults. Values are expressed as the means ± SEMs. ANOVA was used to compare differences between the three interventions. Different superscript letters indicate significant differences between the interventions for oleanolic acid (^a,b^) and for maslinic acid (^c,d^). *p <* 0.05 was considered significant. FOO, functional olive oil; OVOO, optimized virgin olive oil; VOO, virgin olive oil.

Recoveries of triterpenes in urine were consistent with the olive oils consumed in each intervention. Figure 3 shows urinary triterpenes changes (post-intervention minus pre-intervention data) for each olive oil intervention. The amounts of both triterpenic acids in urine after the FOO intervention were about four times higher than those recovered after the VOO and OVOO interventions (*p =* 0.004 and *p <* 0.001, respectively, for maslinic acid, and *p =* 0.026 and *p <* 0.001, respectively, for oleanolic acid). Urine concentrations of total hydroxytyrosol (the sum of hydroxytyrosol and its glucuronide and sulfate conjugates) were higher after the OVOO (2444 mM, *p =* 0.003) and FOO (2876 mM, *p =* 0.002) interventions than after the VOO (115 mM, *p =* 0.011) intervention.

## 4. Discussion

The NUTRAOLEUM study is the first human nutritional clinical trial concerning the effects of VOO that is high in phenolic compounds and enriched with triterpenes, maslinic and oleanolic acids, from olive exocarp, on MS features and endothelial function risk biomarkers in comparison with standard VOO in healthy subjects.

The PREDIMED study reported that a MD supplemented with at least 50 g of dietary VOO caused a reversion of MS after a median follow-up of 4.8 years [25], reducing the rate of CVD events by 30% compared with a low-fat diet control group [26]. In addition, this MD enriched with VOO and without energy restrictions reduced the diabetes risk among individuals at high cardiovascular risk [27]. Consumption of VOO close to 2.7, 164 or 366 ppm/day of phenolic compounds in humans, a 0.03% of hydroxytyrosol in a rodent model, and 50 ppm/day of hydroxytyrosol in a murine model have been reported to improve the blood lipid profile, although results in mice were not conclusive [28], possibly due to differences in the phenolic content of the olive oil and the physio-pathology of the studied animals. Polyphenols from olive oils have been shown to provide additional benefits on HDLc, other than those provided by the MUFA content. However, contradictory data exists on these benefits. In 2015, a meta-analysis reported no effect on HDLc concentration after the intake of VOO with at least 150 ppm of phenolic compounds [29], while, in accordance with our results, a recent systematic review [30] concluded that plasma HDLc was increased in different studies consuming from 2.28 to 75 g/day of olive oil. In the Eurolive Study, a European multicenter study, three olive oils (refined, medium and high) differing in their phenolic compound content (2.7, 164 and 366 ppm/day of phenolic content, respectively) increased HDLc and decreased triacylglycerol concentrations [16]. The increase in HDLc was linear with the olive oil polyphenol content. This additional benefit has also been described in healthy and hypercholesterolemic subjects treated with polyphenol-enriched VOO [31] when excluding patients treated with hypolipidemic medication. Additionally, the increase of olive oil polyphenols in the lipoprotein fraction may increase HDL size, stability, and antioxidant status [32]. It has been reported that the daily intake of olive oils enriched with its own polyphenols (250 ppm or 500 ppm), as well as the intake of olive oil enriched in polyphenols from thyme (250 ppm/day) decrease LDLc and improve the lipoprotein subclass distribution and associated ratios [33]. Other authors have described that a one-year intervention with a MD enriched with VOO improved several LDL characteristics related to its atherogenicity (resistance against oxidation, size, composition, and cytotoxicity) but did not modify the plasma LDLc concentrations in a subsample of subjects at high cardiovascular risk in the PREDIMED study [34]. Our results are consistent with this null effect of VOO on LDLc. On the other hand, besides the weak increase in plasma triacylglycerols observed after interventions with both VOO and OVOO, this was without clinical significance given that levels were low at baseline and remain low at the end of the three interventions, and thus it did not increase cardiovascular risk in these healthy subjects (triacylglycerols < 150 mg/dL) [35]. Our results are in contrast with the triacylglycerol improvement reported in the EUROLIVE Study [16]. A recent meta-analysis and systematic review focused on high polyphenol VOO concluded no effect on plasma triacylglycerol concentrations [29]; in addition, Saibandith et al. [36] stated that the effect of olive oil polyphenols on plasma triacylglycerols remained unclear. However, longer studies are needed to clarify this issue.

To the best of our knowledge, this is the first clinical trial evaluating the effect of olive oil triterpenes on plasma lipids and endothelial function biomarkers. Previous animal studies have reported different effects of oleanolic acid on plasma lipids, depending on the experimental animal model [37]. Based on our results, FOO enriched with triterpenes did not modify plasma HDLc, LDLc, or triacylglycerols, but increased plasma total cholesterol concentrations, although no inter-group significance was found. However, although these modifications do not represent an increase in cardiovascular risk since the values are low enough to be considered safe (total cholesterol < 190 mg/dL) [35], further human clinical trials are needed to demonstrate the potential benefit of olive phenolic compounds and triterpenes on plasma lipid concentrations over longer intervention periods.

Endothelial dysfunction is a critical early event in the development of atherosclerosis [38]. An imbalance between vasodilating and vasoconstricting molecules, such as nitric oxide and endothelin-1, respectively, contributes to the pathogenesis of hypertension and its complications [39]. Our results indicate that plasma endothelin-1 levels decreased after the VOO, OVOO and FOO interventions, and these results were also confirmed throughout ex vivo blood culture experiments. Although endothelin-1 is mainly produced from vascular endothelial cell, several studies have suggested that blood cells, such as polymorphonuclear neutrophils [40] and T-Cells [41], and also macrophages [42] are responsible from circulating levels of endothelin-1. In addition, ex vivo experiment has demonstrated the release of the mature peptide after LPS and LPS + PMA stimulation [40]; Mencarelli et al. [43] have confirmed these results. As demonstrated here, endothelin-1 production was decreased after the three interventions. This effect may be beneficial for cardiovascular risk affected people, since the effect of endothelin-1 has been documented on endothelial and inflammatory cells, which contributes to pathophysiological processes such as vascular hypertrophy, cell proliferation, fibrosis and inflammation [44,45,46]. In humans, the consumption of VOO has shown benefits on blood pressure and endothelial function [47]. In agreement with our results, a meta-analysis stated that olive oils with at least 150 ppm of phenolic compounds exert a moderate effect on lowering SBP and no effects on DBP [29]. Regarding triterpenes, besides the weak SBP increase observed after this intervention, no clinical significance was observed as blood pressure remained under 130 mmHg and did not increase cardiovascular risk [35]. It is reported that SBP varies to a greater degree than DBP [48,49]. For this reason, we calculated pulse pressure and found no significant effect of FOO. A beneficial effect of triterpenes on endothelial function [50] and blood pressure [51] has been described in animal models of hypertension, but further studies are required to explore the mechanisms involved in the effect of specific components of VOO on endothelin-1 regulation.

In vitro studies showed that minor olive oil components, specifically hydroxytyrosol and its metabolites, down-regulate the secretion of E-selectin, *p*-selectin, sICAM-1, and sVCAM-1, affecting endothelial function [52]. However, and in agreement with our results, no differences in sICAM-1 or sVCAM-1 plasma concentrations were reported after 50 mL of VOO or refined olive oil consumption containing (161 or 14.67 ppm/day of phenolic compounds, respectively) in coronary heart disease patients [53]. Another study found a significant reduction in sICAM-1 but not in sVCAM-1 concentrations after the intake of 50 mL/day of olive oil [54]. Recently, it has been proposed that the intake of 25 mL/day of VOO containing 366 ppm of phenolic compounds modulates the expression of several genes related to the renin-angiotensin-aldosterone system [55]. Therefore, further longer studies are needed to reach final conclusions about the effect of VOO minor compounds on molecules modulating endothelial function.

One of the limitations of the present study is that young and healthy subjects are recruited as target population. The present intervention does not appear sufficient to draw definitive conclusions. Therefore, new studies in older subject affected by metabolic syndrome and endothelial dysfunction would be interesting in order to evaluate the beneficial effect of VOO components. Although no differences in dietary intakes were observed during the three interventions, measurements of dietary intake relied on self-reporting and were therefore subjective. Another limitation of this study is that dietary records for the washout periods were not recorded, thus we cannot analyzed dietary intakes during these periods. In addition, our subjects live in the south of Spain, where MD and VOO are highly consumed. Therefore, a 3-week intervention is not long enough to cause significant changes. Further studies are required to find conclusions related to the bioactive compounds presents in olive oils, and to explore the mechanisms involved in these effects of specific components of VOO on MS and endothelial function.

## 5. Conclusions

In conclusion, olive oil rich in polyphenols increased HDLc levels in females, although no differences were found at the end of the three interventions, and improved an endothelial function biomarker both in vivo and ex vivo. No additional benefits were obtained from triterpenes VOO enrichment after 3-wk supplementation. However, further longer studies are warranted on olive oil triterpenes and their health benefits in older subjects particularly in those affected by metabolic syndrome and endothelial dysfunction.

## Figures and Tables

**Table 1:** Characteristics of the administered olive oils.

	VOO	OVOO	FOO
Fatty Acid Profile (%)			
C18:0	2.3	2.2	2.1
C18:1n9	78.9	78.2	78.4
C18:2n6	6.6	6.8	6.9
C18:3n3	0.6	0.7	0.7
C20:0	0.4	0.4	0.4
C20:1	0.3	0.4	0.4
C22:0	0.1	0.1	0.1
C24:0	<0.1	<0.1	<0.1
Total phenolic compounds (ppm)	124	490	487
Hydroxytyrosol and derivates	105	424.0	423.0
Lignanes	18.2	61.3	59.2
Flavonoids	0.7	3.4	3.2
Simple phenols	0.0	0.9	0.9
Total triterpenes (mg/kg)	86.5	86.3	388.8
Maslinic acid	47.3	47.3	217.7
Oleanolic acid	39.2	39.1	171.1
Ursolic acid	<10	<10	<10
α-tocopherol (ppm)	174	183	176
Squalene (mg/100 g)	529.2	536.2	545.5
Total pigments (ppm)	15.73	17.59	16.78
Total carotenoid pigments (ppm)	7.08	6.79	6.97
Total sterols (ppm)	1437	1396	1460

FOO, functional olive oil; OVOO, optimized virgin olive oil; VOO, virgin olive oil.

**Table 2:** Clinical and biochemical characteristics of subjects at baseline according to olive oil administration sequence.

Characteristics	Sequence 1	Sequence 2	Sequence 3
Age, years	32 ± 2	29 ± 2	28 ± 2
Gender, male *n* (%)	12 (60)	10 (53)	8 (42)
BMI, kg/m^2^	24 ± 1	24 ± 1	24 ± 1
Waist circumference, cm	80 ± 2	78 ± 3	77 ± 2
Males	80 ± 3	82 ± 4	82 ± 2
Females	81 ± 5	73 ± 3	73 ± 2
HDLc, mg/dL	58 ± 2	58 ± 3	59 ± 2
Males	55 ± 3	51 ± 3	52 ± 3
Females	64 ± 2	65 ± 5	64 ± 3
LDLc, mg/dL	117 ± 9	107 ± 7	102 ± 5
Total cholesterol, mg/dL	192 ± 10	180 ± 7	175 ± 7
Triacylglycerols, mg/dL	87 ± 7	81 ± 13	67 ± 5
Glucose, mg/dL	90 ± 2	92 ± 2	87 ± 2
Adiponectin, mg/L	11.40 ± 1.38	12.49 ± 1.69	17.20 ± 2.78
Resistin, µg/L	16.38 ± 1.88	16.57 ± 1.82	15.26 ± 1.69
SBP, mmHg	121 ± 2	120 ± 3	118 ± 3
DBP, mmHg	77 ± 2	74 ± 2	71 ± 2
Pulse pressure, mmHg	44 ± 2	46 ± 3	47 ± 2
Endothelin-1, pg/mL	1.35 ± 0.08	1.36 ± 0.10	1.38 ± 0.12
sICAM, ng/mL	74.48 ± 4.50	62.26 ± 3.16	66.10 ± 4.63
sVCAM, ng/mL	459 ± 21	459 ± 30	443 ± 25

Values are expressed as the means ± SEMs. ANOVA and χ^2^ tests were used to compare results between groups. Sequence 1: OVOO, VOO and FOO olive oil, *n* = 20; Sequence 2: VOO, FOO and OVOO olive oil, *n* = 19; Sequence 3: FOO, OVOO and VOO olive oil, *n* = 19. BMI, body mass index; DBP, diastolic blood pressure; FOO, functional olive oil; HDLc, high density lipoprotein cholesterol; LDLc, low density lipoprotein cholesterol; *n*, number of observations; OVOO, optimized virgin olive oil; SBP, systolic blood pressure; SEM, standard error of the mean; sICAM-1, soluble intercellular adhesion molecule; sVCAM-1, soluble vascular cell adhesion molecule; VOO, virgin olive oil.

**Table 3:** Average daily energy and selected nutrient intake of subjects at baseline according to olive oil administration sequence.

Nutritional Characteristics	Sequence 1	Sequence 2	Sequence 3
Energy, kcal	1976 ± 90	2151 ± 138	2074 ± 109
Total carbohydrates, g	200 ± 10	213 ± 16	214 ± 13
Proteins, g	80 (16–222)	96 (37–258)	93 (21–215)
Total fat, g	86 ± 4	102 ± 10	89 ± 6
MUFA, g	31 (12–80)	33 (6–85)	33(6–89)
PUFA, g	13 (3–43)	11 (1–44)	14 (2–49)
SFA, g	21 (6–64)	27 (11–80)	26 (4–66)
Vitamin A, µg retinol equivalents	440 (111–1552)	512 (109–158)	539 (7–1669)
Vitamin C, mg ascorbic acid	70 (4–400)	88 (11–428)	81 (3–335)
Vitamin D, µg	2 (0–41)	2 (0–40)	2 (0–34)
Vitamin E, mg α-tocopherol equivalents	9 (2–29)	9 (1–67)	11 (1–39)
Cholesterol, mg	284 ± 26	354 ± 31	287 ± 24
Alcohol, g	0 (0–52)	0 (0–48)	0 (0–84)
Selenium, µg	31 (5–130)	34 (1–114)	36 (3–117)

Values are expressed as the means ± SEMs or as medians (range). ANOVA was used to compare results between groups for those variables that followed normality, and the Kruskal Wallis test was used for those that did not. Data of 51 subjects were obtained from the 3-day dietary record at baseline. Sequence 1: OVOO, VOO and FOO olive oil, *n* = 54; Sequence 2: VOO, FOO and OVOO olive oil, *n* = 42; Sequence 3: FOO, OVOO and VOO olive oil, *n* = 57. FOO, functional olive oil; MUFA, monounsaturated fatty acids; *n*, number of observations; OVOO, optimized virgin olive oil; PUFA, polyunsaturated fatty acids; SEM, standard error of the mean; SFA, saturated fatty acids; VOO, virgin olive oil.

**Table 4:** Average daily energy and selected nutrient intake of subjects after the three olive oil interventions.

Nutritional Characteristics	VOO	OVOO	FOO
Energy, kcal	1983 (873–4342)	2006 (697–4561)	1914 (780–3457)
Total carbohydrates, g	199 (47–408)	203 (30–531)	175 (38–533)
Proteins, g	77 (24–180)	80 (17–200)	73 (25–207)
Total fat, g	89 (27–244)	94 (24–257)	91 (20–227)
MUFA, g	44 (8–119)	44 (8–113)	45 (9–123)
PUFA, g	11 (4–37)	12 (4–36)	13 (3–41)
SFA, g	25 (7–74)	27 (6–82)	24 (5–69)
Vitamin A, µg retinol equivalents	433 (34–1477)	427 (26–1504)	452 (39–1515)
Vitamin C, mg ascorbic acid	48 (0–280)	60 (0–255)	55 (1–305)
Vitamin D, µg	1.5 (0–32)	1.2 (0–82)	1.2 (0–81)
Vitamin E, mg α-tocopherol equivalents	10 (2–30)	11 (3–32)	11 (2–32)
Cholesterol, mg	285 (11–981)	253 (28–853)	272 (15–1011)
Alcohol, g ethanol	0 (0–97)	0 (0–54)	0 (0–78)
Selenium, µg	30 (0–260)	31 (0–198)	31 (1–160)

Values are expressed as median (range). ANOVA was used to compare intakes between interventions. Data for 51 subjects were obtained from the 3-day dietary record at baseline. Data for 51 subjects were obtained from the 3-day dietary record at baseline. FOO, functional olive oil; MUFA, monounsaturated fatty acids; *n*, number of observations; OVOO, optimized virgin olive oil; PUFA, polyunsaturated fatty acids; SFA, saturated fatty acids; VOO, virgin olive oil.

**Table 5:** Metabolic syndrome and endothelial function biomarkers before and after each olive oil intervention in healthy adults.

Characteristics	VOO		OVOO		FOO	
	Pre-Intervention	Post-Intervention	Pre-Intervention	Post-Intervention	Pre-Intervention	Post-Intervention
BMI, kg/m^2^	23.9 ± 0.1	24 ± 0.1	23.9 ± 0.1	24 ± 0.1	24 ± 0.1	24 ± 0.1
Waist circumference, cm	77.4 ± 0.7	77.2 ± 0.7	77.2 ± 0.7	76.8 ± 0.7	77.4 ± 0.7	76.9 ± 0.7
HDLc, mg/dL	58 ± 2	59 ± 2	57 ± 2	60 ± 2 *	58 ± 2	60 ± 2
Males	52 ± 2	53 ± 2	52 ± 2	52 ± 2	52 ± 2	54 ± 2
Females	64 ± 2	65 ± 2	63 ± 2	67 ± 2 *	65 ± 2	66 ± 2
LDLc, mg/dL	105 ± 4	108 ± 4 ^a,b^	104 ± 4	106 ± 4 ^a^	104 ± 4	111 ± 4 ^b^
Total cholesterol, mg/dL	179 ± 5	182 ± 5	177 ± 5	183 ± 5	178 ± 5	186 ± 5 *
Triacylglycerols, mg/dL	72 ± 7	75 ± 7 *	74 ± 7	81 ± 7 *	75 ± 7	75 ± 7
Glucose, mg/dL	91 ± 2	91 ± 2	91 ± 2	91 ± 2	90 ± 2	91 ± 2
Adiponectin, mg/L	13.74 ± 1.7	12.8 ± 1.7	12.48 ± 1.7	13.6 ± 1.71	12.62 ± 1.71	13.74 ± 1.71
Resistin, µg/L	14.1 ± 1.1	14.1 ± 1.1	14.5 ± 1.1	13.8 ± 1.1	13.8 ± 1.1	14.1 ± 1.1
SBP, mmHg	117 ± 3	115 ± 3 *^,a^	119 ± 3	116 ± 3 ^a,b^	114 ± 3	118 ± 3 *^,b^
DBP, mmHg	72 ± 2	72 ± 2	75 ± 2	73 ± 2	72 ± 2	74 ± 2
Pulse pressure, mmHg	45 ± 1	43 ± 1	44 ± 1	42 ± 1	42 ± 1	45 ± 1
Endothelin-1, pg/mL	1.53 ± 0.15	1.38 ± 0.15 *	1.58 ± 0.15	1.41 ± 0.15 *	1.49 ± 0.15	1.35 ± 0.15 *
sICAM-1, ng/mL	68.09 ± 3.19	67.11 ± 3.19	65.17 ± 3.20	67.73 ± 3.19	65.1 ± 3.20	66.7 ± 3.21
sVCAM-1, ng/mL	451 ± 22	443 ± 23	435 ± 22	451 ± 22	431 ± 22	442 ± 23

Values are expressed as the adjusted means ± SEMs. LMM was used to compare pre-intervention vs. post-interventions with each oil data, and data after the three interventions (post-interventions). * Significant differences between pre-intervention vs. post-intervention data within each intervention with the three olive oils. Different superscript letters indicate significant differences between post-intervention results (^a,b^). *p <* 0.05 was considered significant. DBP, diastolic blood pressure; FOO, functional olive oil; HDLc, high density lipoprotein cholesterol; LDLc, low density lipoprotein cholesterol; OVOO, optimized virgin olive oil; SBP, systolic blood pressure; SEM, standard error of the mean; sICAM-1, soluble intercellular adhesion molecule; sVCAM-1, soluble vascular cell adhesion molecule; VOO, virgin olive oil.

## Supplementary Materials

The following are available online at http://www.mdpi.com/2072-6643/10/5/626/s1, Figure S1: Plasma endothelin-1 ex vivo secretion in whole blood cultures from healthy adults before and after 3 weeks of intervention with the three olive oils and PHA, LPS or PMA + IO stimulation. Table S1: CONSORT 2010 checklist of information to include when reporting a randomized trial.

## Appendix A

### Analysis of Triterpenes in Urine

For the analysis of maslinic acid (MA) and oleanolic acid (OA), aliquots of 250 µL of urine were transferred into 15-mL screw-capped glass tubes and spiked with 1 ng/mL of d_3_-OA, 20 µL of β-glucuronidase from *Escherichia coli* and 200 µL of 0.1 M phosphate buffer pH 6.0. After overnight incubation in a water bath at 37 °C, 50 mg of NaHCO_3_/Na_2_CO_3_ (1:2, *w*/*w*) was added to each tube before extraction. The samples were then subjected to a liquid-liquid extraction with 2 mL of methyl *tert*-butyl ether. The mixture was homogenized in a shaker rotator for 20 min and centrifuged at 3500 rpm for 5 min at room temperature. The organic phase was transferred to clean tubes and evaporated (40 °C) under a stream of nitrogen. The extracts were then derivatized with 50 µL of 2-picolylamine (1 μg/μL in ACN). The reaction mixture was incubated for 10 min at 60 °C on a heating block and then dried under a nitrogen stream. Samples were reconstituted in 100 µL of ACN-H_2_O MilliQ grade (1:1). Derivatized OA and MA in urine was quantified using an Acquity UPLC system, (Waters Associates, Milford, MA, USA) for the chromatographic separation, the column was coupled to a triple quadrupole (Quattro Premier) mass spectrometer provided with an orthogonal Z-spray-electrospray interface (ESI) (Waters Associates). Nitrogen was used as the drying and nebulizing gas. The desolvation gas flow was set to approximately 1200 L/h, and the cone gas flow was set to 50 L/h.

Capillary voltages of 3 kV and 2.5 kV were used in positive and negative ionization mode, respectively. The nitrogen desolvation temperature was set to 450 °C, and the source temperature was set to 120 °C. The collision gas was argon, and the flow rate was 0.21 mL/min.

The liquid chromatography separation was performed at 55 °C using an Acquity CSH phenyl-hexyl column (100 mm, 2.1 mm i.d., 1.7 µm) (Waters Associates) operating at a flow rate of 300 µL min^−1^. Water and methanol, both containing formic acid (0.01% *v*/*v*) and ammonium formate (1 mM), were selected as mobile phase solvents. For the target detection of derivatized OA and MA, a gradient program was used to separate the analytes; the percentage of organic solvent was linearly changed as follows: 0 min, 70%; 0.5 min, 70%; 7 min, 98%; 9 min, 98%; 9.5 min, 70%; and 11 min, 70%.
