# Supplementary material for: Effects of Virgin Olive Oils Differing in Their Bioactive Compound Contents on Metabolic Syndrome and Endothelial Functional Risk Biomarkers in Healthy Adults: A Randomized Double-Blind Controlled Trial
Source: Nutrients. 2018 May 16;10(5):626. doi: 10.3390/nu10050626 (PMC5986505; doi:10.3390/nu10050626)
Supplement: Supplementary file 1 [file nutrients-10-00626-s001.zip › supplementary material/CONSORT-2010-Check_NUTRIENTS CV_28_03_2018.doc]

CONSORT 2010 checklist of information to include when reporting a randomised trial*

| Section/Topic | Item No | Checklist item | Reported on page No |
| --- | --- | --- | --- |
| Title and abstract | | | |
|  | 1a | Identification as a randomised trial in the title | Yes |
|  | 1b | Structured summary of trial design, methods, results, and conclusions (for specific guidance see CONSORT for abstracts) | Yes |
| Introduction | | | |
| Background and objectives | 2a | Scientific background and explanation of rationale | Pages 2  Lines 62-86 |
|  | 2b | Specific objectives or hypotheses | Pages 2  Lines 86-93 |
| Methods | | | |
| Trial design | 3a | Description of trial design (such as parallel, factorial) including allocation ratio | Page 3  Lines 121; 105-117 |
|  | 3b | Important changes to methods after trial commencement (such as eligibility criteria), with reasons | No changes |
| Participants | 4a | Eligibility criteria for participants | Page 3  Lines 97-100 |
|  | 4b | Settings and locations where the data were collected | Page 3  Lines 105-107 |
| Interventions | 5 | The interventions for each group with sufficient details to allow replication, including how and when they were actually administered | Page 3-4  Lines 122-147 |
| Outcomes | 6a | Completely defined pre-specified primary and secondary outcome measures, including how and when they were assessed | 1. Pages 5-6  Lines 182-193  2. Page 6  Lines 198-202 |
|  | 6b | Any changes to trial outcomes after the trial commenced, with reasons | No changes |
| Sample size | 7a | How sample size was determined | Page 3  Lines 96-97 |
|  | 7b | When applicable, explanation of any interim analyses and stopping guidelines | Not applicable |
| Randomisation: |  |  |  |
| Sequence generation | 8a | Method used to generate the random allocation sequence | Page 4  Lines 131-133 |
|  | 8b | Type of randomisation; details of any restriction (such as blocking and block size) | Not applicable |
| Allocation concealment mechanism | 9 | Mechanism used to implement the random allocation sequence (such as sequentially numbered containers), describing any steps taken to conceal the sequence until interventions were assigned | Page 4  Lines 131-133 |
| Implementation | 10 | Who generated the random allocation sequence, who enrolled participants, and who assigned participants to interventions | Page 4  Lines 131-133 |
| Blinding | 11a | If done, who was blinded after assignment to interventions (for example, participants, care providers, those assessing outcomes) and how | Page 4  Lines 134-137 |
|  | 11b | If relevant, description of the similarity of interventions | Not applicable |
| Statistical methods | 12a | Statistical methods used to compare groups for primary and secondary outcomes | Pages 6  Lines 212-231 |
|  | 12b | Methods for additional analyses, such as subgroup analyses and adjusted analyses | Page 6  Lines 232-237 |
| Results | | | |
| Participant flow (a diagram is strongly recommended) | 13a | For each group, the numbers of participants who were randomly assigned, received intended treatment, and were analysed for the primary outcome | Page 3  Lines 108-109; Figure 1 114-117 |
|  | 13b | For each group, losses and exclusions after randomisation, together with reasons | Page 3  Lines 107-110  Figure 1 114-117 |
| Recruitment | 14a | Dates defining the periods of recruitment and follow-up | Page 3 Lines 106-107 |
|  | 14b | Why the trial ended or was stopped | Trial was not stopped |
| Baseline data | 15 | A table showing baseline demographic and clinical characteristics for each group | Table 2 |
| Numbers analysed | 16 | For each group, number of participants (denominator) included in each analysis and whether the analysis was by original assigned groups | Table 2, Table 3, Table 4 |
| Outcomes and estimation | 17a | For each primary and secondary outcome, results for each group, and the estimated effect size and its precision (such as 95% confidence interval) | Pages 7-12 Table 5 |
|  | 17b | For binary outcomes, presentation of both absolute and relative effect sizes is recommended | No applicable |
| Ancillary analyses | 18 | Results of any other analyses performed, including subgroup analyses and adjusted analyses, distinguishing pre-specified from exploratory | Not applicable |
| Harms | 19 | All important harms or unintended effects in each group (for specific guidance see CONSORT for harms) | Not applicable |
| Discussion | | | |
| Limitations | 20 | Trial limitations, addressing sources of potential bias, imprecision, and, if relevant, multiplicity of analyses | Page 14  Line 414-419 |
| Generalisability | 21 | Generalisability (external validity, applicability) of the trial findings | Page 12  Lines 329-332 |
| Interpretation | 22 | Interpretation consistent with results, balancing benefits and harms, and considering other relevant evidence | Yes |
| Other information | | |  |
| Registration | 23 | Registration number and name of trial registry | Abstract; Page 2 Line 56; Page 4 line 146-147 |
| Protocol | 24 | Where the full trial protocol can be accessed, if available | clinicaltrials.gov |
| Funding | 25 | Sources of funding and other support (such as supply of drugs), role of funders | Acknowledgements |

*We strongly recommend reading this statement in conjunction with the CONSORT 2010 Explanation and Elaboration for important clarifications on all the items. If relevant, we also recommend reading CONSORT extensions for cluster randomised trials, non-inferiority and equivalence trials, non-pharmacological treatments, herbal interventions, and pragmatic trials. Additional extensions are forthcoming: for those and for up to date references relevant to this checklist, see [www.consort-statement.org](http://www.consort-statement.org/).
